# Comprehensive analytical and clinical evaluation of a RNA extraction-free saliva-based molecular assay for SARS-CoV-2

**DOI:** 10.1371/journal.pone.0268082

**Published:** 2022-05-05

**Authors:** Joost P. H. Schoeber, Juliëtte M. Schlaghecke, Britt M. J. Meuwissen, Mara van Heertum, Adriaan J. C. van den Brule, Anne J. M. Loonen

**Affiliations:** 1 Research Group Applied Natural Sciences, Fontys University of Applied Sciences, Eindhoven, The Netherlands; 2 Pathologie-DNA, Lab for Molecular Diagnostics, Location Jeroen Bosch Hospital, ’s-Hertogenbosch, The Netherlands; University of Helsinki: Helsingin Yliopisto, FINLAND

## Abstract

Standard SARS-CoV-2 testing protocols using nasopharyngeal/throat (NP/T) swabs are invasive and require trained medical staff for reliable sampling. In addition, it has been shown that PCR is more sensitive as compared to antigen-based tests. Here we describe the analytical and clinical evaluation of our in-house RNA extraction-free saliva-based molecular assay for the detection of SARS-CoV-2. Analytical sensitivity of the test was equal to the sensitivity obtained in other Dutch diagnostic laboratories that process NP/T swabs. In this study, 955 individuals participated and provided NP/T swabs for routine molecular analysis (with RNA extraction) and saliva for comparison. Our RT-qPCR resulted in a sensitivity of 82,86% and a specificity of 98,94% compared to the gold standard. A false-negative ratio of 1,9% was found. The SARS-CoV-2 detection workflow described here enables easy, economical, and reliable saliva processing, useful for repeated testing of individuals.

## Introduction

The coronavirus disease 2019 (COVID-19) pandemic caused by severe acute respiratory syndrome coronavirus 2 (SARS-CoV-2) already resulted in over 273 million infections and more than 5,3 million deaths worldwide (WHO Coronavirus (COVID-19) Dashboard, December 21^st^ 2021, https://covid19.who.int). Molecular diagnostic tests are the gold standard to detect coronavirus. Most frequently, RNA extraction is performed on nasopharyngeal/throat (NP/T) swab samples, followed by reverse transcriptase quantitative PCR (RT-qPCR). Economical, easy to use, sensitive and high throughput assays are of crucial importance during the pandemic to timely diagnose COVID-19 cases, to further prevent transmission in the community. Saliva has been demonstrated to be a good alternative specimen for molecular SARS-CoV-2 diagnostics as it can be self-sampled and shows comparable sensitivity to NP/T swabs [1–3]. Many real-time PCR assays for detection of SARS-CoV-2 have been published [4–6], however, those methods generally apply RNA extraction and purification prior to amplification of (one or multiple) target genes. Here we report the analytical and clinical performance of our RNA extraction-free saliva-based molecular assay, which can be an inexpensive and simple tool to monitor SARS-CoV-2 in (a)symptomatic individuals.

## Materials and methods

### Ethics statement

The Daily Board of the Medical Ethics Committee (Máxima MC, Eindhoven/Veldhoven, The Netherlands) reviewed the research proposal (N21.021) and concluded that the rules laid down in the Medical Research Involving Human Subjects Act (also known by its Dutch abbreviation WMO), did not apply. Written informed consent was obtained from every participant in this study.

### Dutch reference panels

Different analytical samples were provided by the National Institute for Public Health and Environment (RIVM, Bilthoven, The Netherlands), the Dutch reference institute. SARS-CoV-2 (hCoV-19/Netherlands/NoordBrabant_10003/2020) in MEM with HANKs’ salts was heat inactivated at 60°C for 2 hours and had a concentration of 5.62*10^5^ TCID50/mL (1.73*10^8^ digital copies of RdRp-gene and 1.28*10^8^ digital copies of E-gene). Isolation of 200 μL SARS-CoV-2 on MagNApure, elution in 50 μL and 5 μL in PCR results in Ct values of 16.86 for RdRp target and 16.97 for E target.

The specificity panel consisted of simulated clinical samples (EQA6_CoV20-01 –EQA6_CoV20-10) containing hCoV-NL63, hCoV-229e, hCoV-OC43, SARS-CoV-2 (different concentrations) and Influenza virus A (H3N2).

A saliva panel spiked with different concentrations of SARS-CoV-2 was used to determine the analytical sensitivity of the RT-qPCR assay.

### Collection and processing of saliva from symptomatic and asymptomatic individuals

Fresh saliva and NP/T swabs were collected from symptomatic individuals, after informed consent, who visited SARS-CoV-2 testing facilities of Municipal Health Services (MHS) in the period February-April 2021. Study participants were asked to supply a minimum of 500 μL saliva (after not consuming food or drinks (other than plain water) for at least 30 min.) in a 15 or 50 mL tube (Greiner Bio-One B.V., Alphen a/d Rijn, The Netherlands). NP/T swabs (995 samples) were send to routine diagnostic laboratories were RNA extraction was performed followed by RT-qPCR. Fresh self-collected saliva from 347 asymptomatic individuals was processed according to the method described by Ranoa *et al*. [2]. In short, saliva was heat inactivated for 30 min (95°C) and subsequently diluted at a 1:1 ratio with stabilization buffer TM4 (2Wave Diagnostics, Helmond, The Netherlands). Saliva-TM4 samples were directly used in RT-qPCR. Molecular analysis was performed within 24 hours after sample collection and processing for PCR.

### RT-qPCR assay

For the symptomatic group we performed singleplex RT-qPCR assays targeting N1 and Rpp30 (cellularity control). RT-qPCR multiplex assays (*N1* and *Rpp30*) were used for asymptomatic individuals. Primers and probes for both targets were previously published by the Centre for Disease Control and Prevention (CDC, Real-time RT-PCR Primers and Probes for COVID-19 | CDC): *N1* forward primer 5’-GACCCCAAAATCAGCGAAA-3’, reverse primer 5’-TCTGGTTACTGCCAGTTGAATCTG-3’ and probe 5’-FAM-ACCCCGCAT-ZEN-TACGTTTGGTGGACC-3IABkFQ-3’; *Rpp30* forward primer 5’-AGATTTGGACCTGCGAGC G-3’, reverse primer 5’- GAGCGGCTGTCTCCACAAGT-3’ and probe 5’-ATTO647-TTCTGACCT-ZEN-GAAGGCTCTGCGCG-3IABkFQ-3’ (Integrated DNA Technologies, Leuven, Belgium). Periodic *in silico* assessment of *N1* primers and probe was performed to evaluate their specific binding to upcoming SARS-CoV-2 strain variants in The Netherlands (*e*.*g*. Delta (B.1.617.2) and Omicron). During validation, we optimized our workflow for SARS-CoV-2 detection in saliva.

RT-qPCR reactions were prepared with 4x Taqman Fast Virus 1-step (Thermo Fisher Scientific, Brussels, Belgium), 500nM of each primer, 125nM (FAM-labeled) or 75nM (ATTO 647-labeled) probe, and 10 μL of saliva-TM4 sample to an end volume of 22 μL. All RT-qPCR reactions were performed in 0.2mL 96-well plates using a CFX96 touch Real-Time PCR detection System (Bio-Rad Laboratories, Temse, Belgium). The one-step RT-qPCR program consisted of a reverse transcription step of 5 min at 50°C, followed by 95°C for 20 sec, and then 45 cycles of 95°C for 3 sec, 60°C for 30 sec.

## Data analysis

RT-qPCR data from NP/T swabs were provided to our laboratory as Ct-values. PCR data from the CFX96 system were processed using Bio-Rad CFX Maestro software (version 2.0).

Threshold for fluorescent labels was set at 5% of maximum RFU. *Rpp30* cellularity control needed to be in the Ct23-33 range; however, positive *N1* signal overruled this criterium. *N1* target was determined as “positive” at Ct<35.

Sensitivity, specificity, and false negative ratio were calculated using NP/T swab results as the gold standard.

## Results

### *In silico* analysis primers and probe SARS-CoV-2

Periodic evaluation of the used primers and probe sequences for SARS-CoV-2 strain variants did not reveal mismatches with respect to data from the “Global Initiative on Sharing All Influenza Data” (www.GISAID.org) and UCSC Genomce Browser (https://genome.ucsc.edu/covid19.html) during the time-frame of our study; for example for Delta (B.1.617.2) and Omicron variants.

### Performance testing using Dutch reference panels

Analytical validation of the molecular assay consisted of the performance of (*i*.) specificity panel (10 samples); (*ii*.) a spiked saliva panel (6 samples; a negative sample, and *RdRp* target ranging from 4.56*10^1^ to 4.56*10^5^ digital copies/mL); and (*iii*.) a confirmation panel (15 clinical saliva samples from symptomatic individuals (5 positive)).

All samples from the specificity panel were correctly identified; only the four samples containing SARS-CoV-2 virus were positive for the *N1* gene target. Using the spiked saliva panel, analytical sensitivity of the test resulted in 456 digital *RdRp* gene copies/mL, which is equal to the sensitivity obtained in other Dutch diagnostic laboratories that process NP/T swabs and apply RNA extraction workflows. RIVM processed 15 randomly selected clinical saliva samples from our study cohort, using their workflow (RNA extraction followed by Corman PCR [4]), and confirmed the results. Together, this allowed us to perform SARS-CoV-2 diagnostics using saliva (see online repository (https://doi.org/10.34894/GH7WKV) for results of performance of our workflow on spiked saliva samples from RIVM, and the certificate obtained).

### Test performance with symptomatic individuals

**Fig 1:**
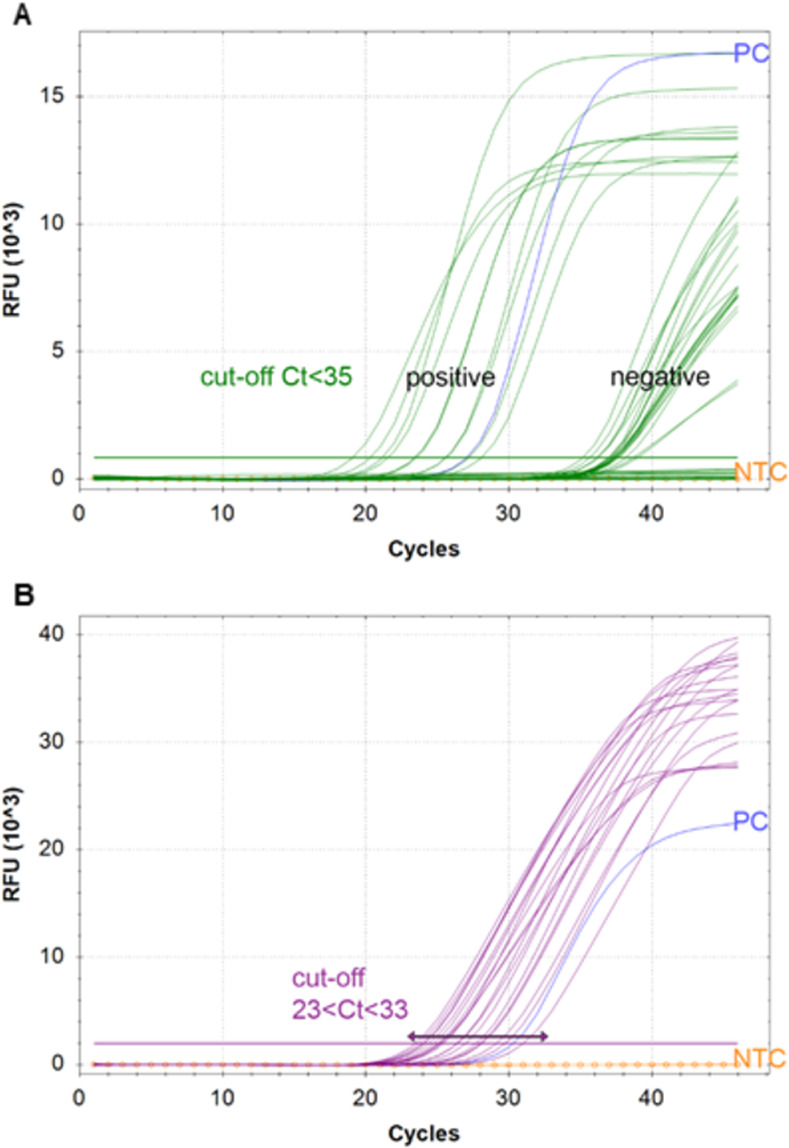
Typical results of *N1* and *Rpp30* amplification after saliva processing. In A FAM channel results are depicted (*N1*). SARS-CoV-2 positive samples show Ct values <35, negative samples Ct>35 or no amplification. In B, ATTO647 channel results are displayed demonstrating the amplification range of Ct 23–33. PC: positive control; NTC: no template control.

In total, 955 symptomatic individuals visiting different MHS testing facilities provided both NP/T swabs and self-collected saliva samples for analysis. NP/T swabs were distributed to different nationally appointed diagnostic laboratories. Processing of the NP/T swabs in those laboratories included RNA extraction and one-step RT-qPCR. MHS provided Ct values of the PCR tests (“gold standard”). Saliva samples were heat inactivated, 1:1 diluted in TM4 stabilization buffer, and directly used in one-step RT-qPCR for *N1* and *Rpp30* targets. Our RT-qPCR resulted in a sensitivity of 82,86% (CI_95%_: 74,27–89,51%) and a specificity of 98,94% (CI_95%_: 98,00–99,51%) compared to the gold standard with predicted prevalence of 10% (see Table 1). A false-negative ratio of 1,9% was found. An example qPCR plot of results obtained during clinical validation, demonstrating magnitude of fluorescence during amplification for both *N1* and *RPP30* gene targets, is shown in Fig 1.

**Table 1:** Comparison of one-step RT-qPCR results from NP/T swabs and saliva samples.

		Gold standard (NP/T swab + PCR)		
		positive	negative	
**Saliva PCR**	**positive**	87	9	96
	**negative**	18	841	859
		105	850	955

### Test performance and follow-up of asymptomatic individuals

From 347 asymptomatic individuals saliva samples were collected and tested for the presence of SARS-CoV-2. Four persons (1,2%) were tested positive, with Ct values ranging from 24,57 to 34,61. In this cohort, 149 individuals (42,9%) provided self-collected saliva samples for at least 2 times/week during 4 consecutive weeks. This provided data to monitor sub-clinical presence of SARS-CoV-2 in saliva (Ct>35): 28,2% (42/149) persons were tested positive.

## Discussion and conclusion

The ongoing COVID-19 pandemic has spread rapidly across the world and becomes a leading cause of morbidity and mortality. Timely identification of SARS-CoV-2 infections is of crucial importance to further prevent transmission in the community. In The Netherlands, NP/T swabs remain the gold standard, involving health care professionals for reliable sampling—and only possible at a limited number of nationally assigned locations.

Our workflow for SARS-CoV-2 detection in self-collected saliva was evaluated and approved by the RIVM and has the potential to increase testing capacity. It also eliminates the need for RNA extraction, saving time and resources.

To our knowledge, this is one of the largest clinical studies comprising both symptomatic (n = 955) and asymptomatic (n = 347) individuals, in The Netherlands. Our workflow for SARS-CoV-2 detection in self-collected saliva from symptomatic individuals visiting different MHS testing facilities identified positive persons with a sensitivity of 82,9% and a specificity of 98,9%, when compared to the described gold standard NP/T swab processing. The rather low sensitivity might result from RNA degradation in a number of samples, or may result from variation in sampling and/or sample transport conditions in this initial study cohort. During validation, we identified critical control points (including logistics) and optimized our workflow for SARS-CoV-2 detection in saliva. As such, saliva has been frequently evaluated as suitable sample for molecular diagnostics, *e*.*g*. Czumbel *et al*. [7] and Sakanashi *et al*. [8]. Considering the conclusions of the European Centre for Disease Prevention and Control [9] stating that “the reported heterogeneity is likely to, in part, reflect differences in sampling techniques, sampling times and the type of population being tested”, we confirm the importance of correct sample handling and logistics (especially because in this study analyses of NP/T swabs and saliva were performed at different diagnostics laboratories). That is, in our workflow, the full 30 min. heat inactivation step—prior to adding the stabilization buffer—showed to be of crucial importance to (help to) prevent viral RNA degradation. Moreover, heat inactivation prior to addition of the stabilization buffer TM4 was chosen for ease of use for individuals (sample collection in an empty tube), prevention of cross-contamination (inactivation of sample in a closed collection tube), and to assure correct sample/TM4 ratio (pipetting equal volumes of sample and 2x stabilization buffer).

In our study cohort, 18 individuals were tested negative for the presence of SARS-CoV-2 in saliva compared to the gold standard (NP/T swab workflow). It has to be noted that 5 of the 18 samples resulted in an amplification curve for *N1* target with onset after Ct 35. We critically evaluated the remaining 13 samples, to further improve our methodology. First, samples were re-analysed, giving similar results. Second, total RNA extraction was performed after spiking with Phocine Distemper Virus (PDV), followed by RT (using random hexamers) and qPCR targeting either PDV (external) or Porphobilinogen Deaminase (PBGD) (internal) control cDNA sequences, investigating possible RNA degradation or inhibition. Third, 5 randomly selected samples were sequenced for their *N1* PCR targets, to investigate the presence of mutations that may result in insufficient amplification of those loci. None of the experiments provided clear answers to why the ‘saliva workflow’ showed discrepant results compared to the NP/T swab workflow. Next, we differentiated the data based on, *i)* sampling date, *ii)* macroscopic characteristics, and *iii)* the MHS test location (and subsequent appointed diagnostic laboratory). Macroscopic characteristics included sample color (dark–very dark samples), visible food particles in sample and viscosity. When comparing samples analysed by MHS laboratory 1 (n = 144 samples) a sensitivity and specificity of 90% and 96% was found, respectively. MHS laboratory 2 analysed 811 NP/T samples and comparison resulted in a sensitivity and specificity of 82% and 99%, respectively. In total, 17/18 discrepant results came from MHS laboratory 2. Positive NP/T samples from MHS laboratory 1 had an average Ct value of 23,70 (median Ct 22,20) and samples from laboratory 2 showed an average Ct value of 22,15 (median Ct 20,93). Our saliva workflow identified an additional 9 positives, that were missed by NP/T swab analysis. These 9 samples were equally distributed over both MHS diagnostics laboratories, and it supports that a 100% match between the different methods for analysis or performing laboratories is unlikely to occur. RIVM evaluates the performance of the appointed Dutch diagnostic laboratories by comparing results obtained from sensitivity, specificity and confirmation verification panels, and although all approved by RIVM, laboratories may use different testing methods and self-defined cut-off levels most likely leading to inter-lab variation.

Currently, both the logistics procedures and the molecular assay were optimized to a quadruplex assay targeting *N1* gene, *E* gene, *Rpp30* (cellularity control) and *PDV* (RT control). A first clinical evaluation using this optimized workflow, investigating NP/T swabs and self-collected saliva of 198 symptomatic individuals, resulted in a sensitivity of 95,56% and a specificity of 100% (https://doi.org/10.34894/GH7WKV).

Taken together, this gives us the confidence that saliva is a suitable sample for SARS-CoV-2 diagnostics, enabling economical, easy and reliable processing and testing from symptomatic individuals, as also shown by others [9–11]. Moreover, it has been shown that the course of infection is altered for Omicron, with higher viral shedding in saliva relative to nasal samples, resulting in improved diagnostics using saliva [12].

Besides symptomatic individuals, we also monitored 347 asymptomatic individuals. In total, 149/347 individuals provided self-collected saliva samples twice a week during 4 consecutive weeks, comprising a unique study cohort with follow-up data. Before collecting saliva samples, the individuals filled in a Coronavirus health check questionnaire (cf. document from RIVM) and stated that they had no clinical symptoms or had been in close contact with corona-positive persons. We found 28,2% of this asymptomatic follow-up cohort to test positive for SARS-CoV-2 with Ct>35, at least once during those 4 weeks. This is in contrast with the large study cohort of symptomatic individuals of which 9,0% (86/955) tested positive for SARS-CoV-2 with Ct>35. Suggestion that those asymptomatic individuals are carriers of SARS-CoV-2 was further supported by the finding that the 4 asymptomatic persons that were tested positive (Ct<35) displayed Ct>35 values at earlier sampling moments in the 4 weeks period.

In conclusion, The SARS-CoV-2 detection workflow described here enables easy and reliable saliva processing and testing, from (a)symptomatic individuals. It also eliminates the need for trained medical staff for reliable NP/T sampling.

## Supporting information

S1 Table Sensitivity panel RIVM. (PDF)

S2 Table Clinical validation Fontys/MHS study. (PDF)

S3 Table Quadruplex qPCR clinical validation. (PDF)
