# Supplementary material for: Comprehensive analytical and clinical evaluation of a RNA extraction-free saliva-based molecular assay for SARS-CoV-2
Source: PLoS One. 2022 May 5;17(5):e0268082. doi: 10.1371/journal.pone.0268082 (PMC9070935; doi:10.1371/journal.pone.0268082)
Supplement: S1 Table — (PDF) [file pone.0268082.s001.pdf]

DOI Table 1. Sensitivity panel RIVM

Sample name	Spiked *RdRP* (digital copies/mL)	Classification RIVM (SARS-CoV-2 positive/negative)	In house molecular assay with *N1* target gene (Ct value) *	Classification in house molecular assay (SARS-CoV-2 positive/negative)	Classification by other Dutch laboratories #
Sen.SALIVA_CoV20-04	$4.56 \cdot 10^5$	positive	27,73	positive	100% (pos.)
Sen.SALIVA_CoV20-01	$4.56 \cdot 10^4$	positive	30,82	positive	100% (pos.)
Sen.SALIVA_CoV20-06	$4.56 \cdot 10^3$	positive	35,05	positive	95% (pos.)
Sen.SALIVA_CoV20-05	$4.56 \cdot 10^2$	positive	38,14**	unequivocal***	40% (pos.)
Sen.SALIVA_CoV20-03	$4.56 \cdot 10^1$	negative	N/A	negative	100% (neg.)
Sen.SALIVA_CoV20-02	no virus: neg. control	negative	N/A	negative	100% (neg.)

SARS-CoV-2 spiked sensitivity panel of the RIVM, standardized by number of *RdRP* copies/mL. * Mean of triplicate measurements; ** 1 of 3 N/A; *** At the time of measuring uncertain whether person would have clinical symptoms or not; # Scoring of saliva sensitivity panel by other Dutch laboratories (percentage of Dutch laboratories with correct identification according to RIVM); N/A = no amplification detected.
