# Supplementary material for: Comprehensive analytical and clinical evaluation of a RNA extraction-free saliva-based molecular assay for SARS-CoV-2
Source: PLoS One. 2022 May 5;17(5):e0268082. doi: 10.1371/journal.pone.0268082 (PMC9070935; doi:10.1371/journal.pone.0268082)
Supplement: S2 Table — (PDF) [file pone.0268082.s002.pdf]

Clinical validation Fontys/MHS study

		sample date	MHS result	MHS Ct	Fontys result	N-target Ct
Fontys pos / MHS neg: 9	Fontys: **Ct < 30** amount: 4	2021-03-22	Negative		positive	23,92
		2021-02-16	Negative		positive	28,73
		2021-02-16	Negative		positive	29,56
		2021-02-16	Negative		positive	29,94
	Fontys: **30 < Ct < 35** amount: 5	2021-02-16	Negative		positive	30,28
		2021-02-16	Negative		positive	30,47
		2021-04-06	Negative		positive	31,16
		2021-04-06	Negative		positive	31,73
		2021-03-22	Negative		positive	32,51
	Fontys: **Ct > 35** amount: 86	2021-03-24	Negative			35,61
		2021-03-24	Negative			35,73
		2021-03-18	Negative			36,16
		2021-03-24	Negative			36,16
		2021-03-24	Negative			36,17
		2021-03-24	Negative			36,17
		2021-03-22	Negative			36,43
		2021-03-22	Negative			36,46
		2021-04-06	Negative			36,47
		2021-02-23	Negative			36,72
		2021-03-22	Negative			36,81
		2021-03-22	Negative			36,89
		2021-02-23	Negative			36,91
		2021-03-24	Negative			36,95
		2021-03-18	Negative			37,01
		2021-03-18	Negative			37,02
		2021-03-18	Negative			37,02
		2021-03-24	Negative			37,03
		2021-03-24	Negative			37,04
		2021-03-22	Negative			37,05
		2021-03-18	Negative			37,05
		2021-03-18	Negative			37,06
		2021-03-24	Negative			37,09
		2021-03-22	Negative			37,10

2021-03-24	Negative	37,14
2021-03-22	Negative	37,17
2021-03-24	Negative	37,19
2021-03-22	Negative	37,19
2021-03-22	Negative	37,22
2021-03-18	Negative	37,22
2021-03-24	Negative	37,23
2021-03-24	Negative	37,24
2021-03-24	Negative	37,25
2021-03-18	Negative	37,27
2021-03-18	Negative	37,28
2021-03-18	Negative	37,31
2021-03-24	Negative	37,33
2021-03-24	Negative	37,34
2021-03-24	Negative	37,35
2021-03-22	Negative	37,37
2021-03-24	Negative	37,37
2021-03-22	Negative	37,38
2021-03-24	Negative	37,42
2021-03-24	Negative	37,47
2021-03-24	Negative	37,48
2021-03-22	Negative	37,50
2021-04-06	Negative	37,51
2021-03-24	Negative	37,51
2021-03-22	Negative	37,53
2021-03-18	Negative	37,60
2021-03-24	Negative	37,64
2021-03-24	Negative	37,64
2021-03-18	Negative	37,67
2021-03-18	Negative	37,74
2021-03-24	Negative	37,77
2021-03-22	Negative	37,78
2021-04-06	Negative	37,79
2021-03-22	Negative	37,79
2021-03-24	Negative	37,86
2021-03-22	Negative	37,88
2021-02-18	Negative	37,90
2021-03-24	Negative	37,95
2021-03-18	Negative	38,01

		2021-04-06	Negative			38,04
		2021-03-22	Negative			38,08
		2021-04-06	Negative			38,08
		2021-03-24	Negative			38,10
		2021-03-24	Negative			38,14
		2021-04-06	Negative			38,15
		2021-03-18	Negative			38,19
		2021-04-06	Negative			38,26
		2021-04-06	Negative			38,32
		2021-03-24	Negative			38,35
		2021-03-24	Negative			38,41
		2021-03-24	Negative			38,42
		2021-04-06	Negative			38,56
		2021-04-08	Negative			38,56
		2021-03-24	Negative			38,65
		2021-03-22	Negative			38,76
		2021-03-22	Negative			38,81
		2021-03-24	Negative			38,97
		2021-04-06	Negative			39,46
		2021-02-23	Negative			39,49
		2021-02-18	Negative			39,75
		2021-04-08	Negative			45,32
		2021-04-08	Negative			45,38
MHS pos / Fontys neg: 18		2021-02-16	Positive	35,02	negative	
		2021-03-18	Positive	34,6	negative	
		2021-03-18	Positive	27,93	negative	
		2021-03-22	Positive	30,11	negative	
		2021-03-22	Positive	24,27	negative	
		2021-03-22	Positive	21,32	negative	
		2021-03-24	Positive	30,68	negative	
		2021-03-24	Positive	35,56	negative	
		2021-04-06	Positive	34,8	negative	
		2021-04-06	Positive	34,7	negative	
		2021-04-06	Positive	30,9	negative	
		2021-04-08	Positive	38	negative	
		2021-04-08	Positive	35,85	negative	
		2021-03-24	Positive	27,97		35,15
		2021-03-18	Positive	20,93		35,42

		2021-03-24	Positive	22,19		36,50
		2021-04-08	Positive	29,41		37,83
		2021-04-06	Positive	30		39,20
	Fontys:	2021-04-08	Positive	17,73	Positive	15,92
	**Ct < 30**	2021-04-06	Positive	21,7	Positive	17,22
Match MHS and Fontys: 87		2021-04-08	Positive	19,48	Positive	17,46
	amount:	2021-03-22	Positive	14,37	Positive	17,58
	61	2021-03-24	Positive	14,7	Positive	18,04
		2021-03-22	Positive	17,5	Positive	18,45
		2021-02-18	Positive	19,61	Positive	18,46
		2021-04-08	Positive	22,1	Positive	18,79
		2021-04-08	Positive	16,56	Positive	18,83
		2021-03-24	Positive	13,98	Positive	19,07
		2021-04-08	Positive	16,61	Positive	19,19
		2021-02-18	Positive	21,61	Positive	20,25
		2021-03-24	Positive	18,09	Positive	20,27
		2021-03-22	Positive	17,44	Positive	20,81
		2021-03-24	Positive	15,76	Positive	21,13
		2021-03-22	Positive	16,37	Positive	21,45
		2021-03-24	Positive	15,91	Positive	21,50
		2021-04-08	Positive	25,91	Positive	21,64
		2021-03-22	Positive	15,15	Positive	22,80
		2021-03-22	Positive	16,35	Positive	23,10
		2021-02-18	Positive	25,84	Positive	23,11
		2021-02-25	Positive	21,94	Positive	23,20
		2021-03-24	Positive	15,03	Positive	23,37
		2021-04-06	Positive	20,9	Positive	23,42
		2021-03-24	Positive	20,61	Positive	23,48
		2021-04-08	Positive	21,47	Positive	23,76
		2021-03-22	Positive	22,47	Positive	24,24
		2021-03-24	Positive	20,27	Positive	24,29
		2021-04-08	Positive	17,28	Positive	24,31
		2021-03-18	Positive	17,75	Positive	24,55
		2021-04-06	Positive	27	Positive	24,74
		2021-02-18	Positive	23,95	Positive	25,12
		2021-04-08	Positive	20,24	Positive	25,15
		2021-03-24	Positive	19,35	Positive	25,54
		2021-03-24	Positive	27,66	Positive	25,57

		2021-03-18	Positive	16,83	Positive	25,97
		2021-03-24	Positive	16,86	Positive	26,43
		2021-03-22	Positive	18,76	Positive	26,79
		2021-03-18	Positive	20,82	Positive	26,90
		2021-04-08	Positive	23,82	Positive	26,93
		2021-02-25	Positive	21,06	Positive	27,08
		2021-03-24	Positive	24,08	Positive	27,17
		2021-04-06	Positive	24,1	Positive	27,22
		2021-03-22	Positive	22,12	Positive	27,28
		2021-02-18	Positive	21,44	Positive	27,49
		2021-04-08	Positive	15,29	Positive	27,50
		2021-04-08	Positive	20,08	Positive	27,56
		2021-04-08	Positive	21,85	Positive	27,75
		2021-04-08	Positive	28,36	Positive	27,83
		2021-03-22	Positive	19,01	Positive	27,85
		2021-04-08	Positive	19,27	Positive	27,94
		2021-03-24	Positive	23,17	Positive	28,07
		2021-04-08	Positive	16,38	Positive	28,38
		2021-04-06	Positive	23,9	Positive	28,54
		2021-03-22	Positive	19,31	Positive	28,84
		2021-04-08	Positive	18,1	Positive	29,36
		2021-03-18	Positive	21,38	Positive	29,38
		2021-03-24	Positive	18,57	Positive	29,57
		2021-03-22	Positive	21,43	Positive	29,65
		2021-03-22	Positive	17,44	Positive	29,79
		2021-03-18	Positive	18,7	Positive	29,86
	Fontys:	2021-03-22	Positive	18,14	Positive	30,10
	**30 < Ct < 35**	2021-04-08	Positive	18,26	Positive	30,15
		2021-03-24	Positive	21,9	Positive	30,24
	amount:	2021-03-22	Positive	18,34	Positive	30,34
	26	2021-02-25	Positive	22,46	Positive	30,59
		2021-04-08	Positive	19,54	Positive	31,18
		2021-03-24	Positive	18,38	Positive	31,27
		2021-04-06	Positive	32,7	Positive	31,33
		2021-03-22	Positive	19,93	Positive	31,83
		2021-03-22	Positive	26,69	Positive	32,01
		2021-03-24	Positive	21,63	Positive	32,03
		2021-02-16	Positive	24,11	Positive	32,10
		2021-03-24	Positive	18,93	Positive	32,18

	2021-03-22	Positive	17,69	Positive	32,27
	2021-03-22	Positive	22,88	Positive	32,31
	2021-03-22	Positive	22,4	Positive	32,43
	2021-03-18	Positive	23,64	Positive	32,52
	2021-03-18	Positive	19,99	Positive	32,60
	2021-04-06	Positive	30,09	Positive	32,63
	2021-03-22	Positive	32,23	Positive	32,85
	2021-04-08	Positive	22,94	Positive	33,40
	2021-03-22	Positive	21,6	Positive	33,48
	2021-03-22	Positive	25,8	Positive	33,91
	2021-03-18	Positive	24,19	Positive	34,09
	2021-04-08	Positive	25,09	Positive	34,15
	2021-03-24	Positive	16,46	Positive	34,18

MHS neg / Fontys neg: 755	2021-04-06	negative		negative	
	2021-04-06	negative		negative	
	2021-04-06	negative		negative	
	2021-02-16	Negative		negative	
	2021-02-16	Negative		negative	
	2021-02-16	Negative		negative	
	2021-02-16	Negative		negative	
	2021-02-16	Negative		negative	
	2021-02-16	Negative		negative	
	2021-02-16	Negative		negative	
	2021-02-16	Negative		negative	
	2021-02-16	Negative		negative	
	2021-02-16	Negative		negative	
	2021-02-16	Negative		negative	
	2021-02-16	Negative		negative	
	2021-02-16	Negative		negative	
	2021-02-16	Negative		negative	
	2021-02-16	Negative		negative	
	2021-02-16	Negative		negative	
	2021-02-16	Negative		negative	
	2021-02-16	Negative		negative	
	2021-02-16	Negative		negative	
	2021-02-16	Negative		negative	
	2021-02-16	Negative		negative	
	2021-02-18	Negative		negative	

		2021-02-18	Negative		negative	
		2021-02-18	Negative		negative	
		2021-02-18	Negative		negative	
		2021-02-18	Negative		negative	
		2021-02-18	Negative		negative	
		2021-02-18	Negative		negative	
		2021-02-18	Negative		negative	
		2021-02-18	Negative		negative	
		2021-02-18	Negative		negative	
		2021-02-18	Negative		negative	
		2021-02-18	Negative		negative	
		2021-02-18	Negative		negative	
		2021-02-18	Negative		negative	
		2021-02-18	Negative		negative	
		2021-02-18	Negative		negative	
		2021-02-18	Negative		negative	
		2021-02-18	Negative		negative	
		2021-02-18	Negative		negative	
		2021-02-18	Negative		negative	
		2021-02-18	Negative		negative	
		2021-02-18	Negative		negative	
		2021-02-18	Negative		negative	
		2021-02-18	Negative		negative	
		2021-02-18	Negative		negative	
		2021-02-18	Negative		negative	
		2021-02-18	Negative		negative	
		2021-02-18	Negative		negative	
		2021-02-18	Negative		negative	
		2021-02-18	Negative		negative	
		2021-02-18	Negative		negative	
		2021-02-18	Negative		negative	
		2021-02-18	Negative		negative	
		2021-02-18	Negative		negative	
		2021-02-18	Negative		negative	
		2021-02-18	Negative		negative	
		2021-02-18	Negative		negative	
		2021-02-18	Negative		negative	
		2021-02-23	Negative		negative	
		2021-02-23	Negative		negative	

		2021-02-23	Negative		negative	
		2021-02-23	Negative		negative	
		2021-02-23	Negative		negative	
		2021-02-23	Negative		negative	
		2021-02-23	Negative		negative	
		2021-02-23	Negative		negative	
		2021-02-23	Negative		negative	
		2021-02-23	Negative		negative	
		2021-02-23	Negative		negative	
		2021-02-23	Negative		negative	
		2021-02-23	Negative		negative	
		2021-02-23	Negative		negative	
		2021-02-23	Negative		negative	
		2021-02-23	Negative		negative	
		2021-02-23	Negative		negative	
		2021-02-23	Negative		negative	
		2021-02-23	Negative		negative	
		2021-02-23	Negative		negative	
		2021-02-23	Negative		negative	
		2021-02-23	Negative		negative	
		2021-02-23	Negative		negative	
		2021-02-23	Negative		negative	
		2021-02-23	Negative		negative	
		2021-02-23	Negative		negative	
		2021-02-23	Negative		negative	
		2021-02-23	Negative		negative	
		2021-02-23	Negative		negative	
		2021-02-23	Negative		negative	
		2021-02-23	Negative		negative	
		2021-02-23	Negative		negative	
		2021-02-23	Negative		negative	
		2021-02-23	Negative		negative	
		2021-02-23	Negative		negative	
		2021-02-23	Negative		negative	
		2021-02-23	Negative		negative	
		2021-02-23	Negative		negative	
		2021-02-23	Negative		negative	
		2021-02-23	Negative		negative	
		2021-02-23	Negative		negative	

2021-02-23	Negative		negative	
2021-02-25	Negative		negative	
2021-02-25	Negative		negative	
2021-02-25	Negative		negative	
2021-02-25	Negative		negative	
2021-02-25	Negative		negative	
2021-02-25	Negative		negative	
2021-02-25	Negative		negative	
2021-02-25	Negative		negative	
2021-02-25	Negative		negative	
2021-02-25	Negative		negative	
2021-02-25	Negative		negative	
2021-02-25	Negative		negative	
2021-02-25	Negative		negative	
2021-02-25	Negative		negative	
2021-02-25	Negative		negative	
2021-02-25	Negative		negative	
2021-02-25	Negative		negative	
2021-02-25	Negative		negative	
2021-02-25	Negative		negative	
2021-02-25	Negative		negative	
2021-02-25	Negative		negative	
2021-02-25	Negative		negative	
2021-02-25	Negative		negative	
2021-03-18	Negative		negative	
2021-03-18	Negative		negative	
2021-03-18	Negative		negative	
2021-03-18	Negative		negative	
2021-03-18	Negative		negative	
2021-03-18	Negative		negative	
2021-03-18	Negative		negative	
2021-03-18	Negative		negative	
2021-03-18	Negative		negative	
2021-03-18	Negative		negative	
2021-03-18	Negative		negative	
2021-03-18	Negative		negative	
2021-03-18	Negative		negative	
2021-03-18	Negative		negative	
2021-03-18	Negative		negative	

2021-03-18	Negative		negative	
2021-03-18	Negative		negative	
2021-03-18	Negative		negative	
2021-03-18	Negative		negative	
2021-03-18	Negative		negative	
2021-03-18	Negative		negative	
2021-03-18	Negative		negative	
2021-03-18	Negative		negative	
2021-03-18	Negative		negative	
2021-03-18	Negative		negative	
2021-03-18	Negative		negative	
2021-03-18	Negative		negative	
2021-03-18	Negative		negative	
2021-03-18	Negative		negative	
2021-03-18	Negative		negative	
2021-03-18	Negative		negative	
2021-03-18	Negative		negative	
2021-03-18	Negative		negative	
2021-03-18	Negative		negative	
2021-03-18	Negative		negative	
2021-03-18	Negative		negative	
2021-03-18	Negative		negative	
2021-03-18	Negative		negative	
2021-03-18	Negative		negative	
2021-03-18	Negative		negative	
2021-03-18	Negative		negative	
2021-03-18	Negative		negative	
2021-03-18	Negative		negative	
2021-03-18	Negative		negative	
2021-03-18	Negative		negative	
2021-03-18	Negative		negative	
2021-03-18	Negative		negative	
2021-03-18	Negative		negative	
2021-03-18	Negative		negative	
2021-03-18	Negative		negative	
2021-03-18	Negative		negative	
2021-03-18	Negative		negative	
2021-03-18	Negative		negative	
2021-03-18	Negative		negative	

	2021-03-18	Negative		negative	
	2021-03-18	Negative		negative	
	2021-03-18	Negative		negative	
	2021-03-18	Negative		negative	
	2021-03-18	Negative		negative	
	2021-03-18	Negative		negative	
	2021-03-18	Negative		negative	
	2021-03-18	Negative		negative	
	2021-03-18	Negative		negative	
	2021-03-18	Negative		negative	
	2021-03-18	Negative		negative	
	2021-03-18	Negative		negative	
	2021-03-18	Negative		negative	
	2021-03-18	Negative		negative	
	2021-03-18	Negative		negative	
	2021-03-18	Negative		negative	
	2021-03-18	Negative		negative	
	2021-03-18	Negative		negative	
	2021-03-18	Negative		negative	
	2021-03-18	Negative		negative	
	2021-03-18	Negative		negative	
	2021-03-18	Negative		negative	
	2021-03-18	Negative		negative	
	2021-03-18	Negative		negative	
	2021-03-18	Negative		negative	
	2021-03-18	Negative		negative	
	2021-03-18	Negative		negative	
	2021-03-18	Negative		negative	
	2021-03-18	Negative		negative	
	2021-03-18	Negative		negative	
	2021-03-18	Negative		negative	
	2021-03-18	Negative		negative	
	2021-03-18	Negative		negative	
	2021-03-18	Negative		negative	
	2021-03-18	Negative		negative	
	2021-03-18	Negative		negative	
	2021-03-18	Negative		negative	
	2021-03-18	Negative		negative	
	2021-03-18	Negative		negative	

	2021-03-18	Negative		negative	
	2021-03-18	Negative		negative	
	2021-03-18	Negative		negative	
	2021-03-18	Negative		negative	
	2021-03-22	Negative		negative	
	2021-03-22	Negative		negative	
	2021-03-22	Negative		negative	
	2021-03-22	Negative		negative	
	2021-03-22	Negative		negative	
	2021-03-22	Negative		negative	
	2021-03-22	Negative		negative	
	2021-03-22	Negative		negative	
	2021-03-22	Negative		negative	
	2021-03-22	Negative		negative	
	2021-03-22	Negative		negative	
	2021-03-22	Negative		negative	
	2021-03-22	Negative		negative	
	2021-03-22	Negative		negative	
	2021-03-22	Negative		negative	
	2021-03-22	Negative		negative	
	2021-03-22	Negative		negative	
	2021-03-22	Negative		negative	
	2021-03-22	Negative		negative	
	2021-03-22	Negative		negative	
	2021-03-22	Negative		negative	
	2021-03-22	Negative		negative	
	2021-03-22	Negative		negative	
	2021-03-22	Negative		negative	
	2021-03-22	Negative		negative	
	2021-03-22	Negative		negative	
	2021-03-22	Negative		negative	
	2021-03-22	Negative		negative	
	2021-03-22	Negative		negative	
	2021-03-22	Negative		negative	
	2021-03-22	Negative		negative	
	2021-03-22	Negative		negative	
	2021-03-22	Negative		negative	
	2021-03-22	Negative		negative	
	2021-03-22	Negative		negative	

	2021-03-22	Negative		negative	
	2021-03-22	Negative		negative	
	2021-03-22	Negative		negative	
	2021-03-22	Negative		negative	
	2021-03-22	Negative		negative	
	2021-03-22	Negative		negative	
	2021-03-22	Negative		negative	
	2021-03-22	Negative		negative	
	2021-03-22	Negative		negative	
	2021-03-22	Negative		negative	
	2021-03-22	Negative		negative	
	2021-03-22	Negative		negative	
	2021-03-22	Negative		negative	
	2021-03-22	Negative		negative	
	2021-03-22	Negative		negative	
	2021-03-22	Negative		negative	
	2021-03-22	Negative		negative	
	2021-03-22	Negative		negative	
	2021-03-22	Negative		negative	
	2021-03-22	Negative		negative	
	2021-03-22	Negative		negative	
	2021-03-22	Negative		negative	
	2021-03-22	Negative		negative	
	2021-03-22	Negative		negative	
	2021-03-22	Negative		negative	
	2021-03-22	Negative		negative	
	2021-03-22	Negative		negative	
	2021-03-22	Negative		negative	
	2021-03-22	Negative		negative	
	2021-03-22	Negative		negative	
	2021-03-22	Negative		negative	
	2021-03-22	Negative		negative	
	2021-03-22	Negative		negative	
	2021-03-22	Negative		negative	
	2021-03-22	Negative		negative	
	2021-03-22	Negative		negative	
	2021-03-22	Negative		negative	
	2021-03-22	Negative		negative	
	2021-03-22	Negative		negative	

		2021-03-22	Negative		negative	
		2021-03-22	Negative		negative	
		2021-03-22	Negative		negative	
		2021-03-22	Negative		negative	
		2021-03-22	Negative		negative	
		2021-03-22	Negative		negative	
		2021-03-22	Negative		negative	
		2021-03-22	Negative		negative	
		2021-03-22	Negative		negative	
		2021-03-22	Negative		negative	
		2021-03-22	Negative		negative	
		2021-03-22	Negative		negative	
		2021-03-22	Negative		negative	
		2021-03-22	Negative		negative	
		2021-03-22	Negative		negative	
		2021-03-22	Negative		negative	
		2021-03-22	Negative		negative	
		2021-03-22	Negative		negative	
		2021-03-22	Negative		negative	
		2021-03-22	Negative		negative	
		2021-03-22	Negative		negative	
		2021-03-22	Negative		negative	
		2021-03-22	Negative		negative	
		2021-03-22	Negative		negative	
		2021-03-22	Negative		negative	
		2021-03-22	Negative		negative	
		2021-03-22	Negative		negative	
		2021-03-22	Negative		negative	
		2021-03-22	Negative		negative	
		2021-03-22	Negative		negative	
		2021-03-22	Negative		negative	
		2021-03-22	Negative		negative	
		2021-03-22	Negative		negative	
		2021-03-22	Negative		negative	
		2021-03-22	Negative		negative	
		2021-03-22	Negative		negative	
		2021-03-22	Negative		negative	
		2021-03-22	Negative		negative	
		2021-03-22	Negative		negative	

	2021-03-22	Negative		negative	
	2021-03-22	Negative		negative	
	2021-03-22	Negative		negative	
	2021-03-22	Negative		negative	
	2021-03-22	Negative		negative	
	2021-03-22	Negative		negative	
	2021-03-22	Negative		negative	
	2021-03-22	Negative		negative	
	2021-03-22	Negative		negative	
	2021-03-22	Negative		negative	
	2021-03-22	Negative		negative	
	2021-03-22	Negative		negative	
	2021-03-22	Negative		negative	
	2021-03-22	Negative		negative	
	2021-03-22	Negative		negative	
	2021-03-22	Negative		negative	
	2021-03-22	Negative		negative	
	2021-03-22	Negative		negative	
	2021-03-22	Negative		negative	
	2021-03-22	Negative		negative	
	2021-03-22	Negative		negative	
	2021-03-22	Negative		negative	
	2021-03-22	Negative		negative	
	2021-03-22	Negative		negative	
	2021-03-22	Negative		negative	
	2021-03-22	Negative		negative	
	2021-03-22	Negative		negative	
	2021-03-22	Negative		negative	
	2021-03-22	Negative		negative	
	2021-03-22	Negative		negative	
	2021-03-22	Negative		negative	
	2021-03-22	Negative		negative	
	2021-03-22	Negative		negative	
	2021-03-22	Negative		negative	
	2021-03-22	Negative		negative	
	2021-03-22	Negative		negative	
	2021-03-22	Negative		negative	
	2021-03-22	Negative		negative	
	2021-03-22	Negative		negative	

2021-03-22	Negative		negative	
2021-03-22	Negative		negative	
2021-03-22	Negative		negative	
2021-03-22	Negative		negative	
2021-03-24	Negative		negative	
2021-03-24	Negative		negative	
2021-03-24	Negative		negative	
2021-03-24	Negative		negative	
2021-03-24	Negative		negative	
2021-03-24	Negative		negative	
2021-03-24	Negative		negative	
2021-03-24	Negative		negative	
2021-03-24	Negative		negative	
2021-03-24	Negative		negative	
2021-03-24	Negative		negative	
2021-03-24	Negative		negative	
2021-03-24	Negative		negative	
2021-03-24	Negative		negative	
2021-03-24	Negative		negative	
2021-03-24	Negative		negative	
2021-03-24	Negative		negative	
2021-03-24	Negative		negative	
2021-03-24	Negative		negative	
2021-03-24	Negative		negative	
2021-03-24	Negative		negative	
2021-03-24	Negative		negative	
2021-03-24	Negative		negative	
2021-03-24	Negative		negative	
2021-03-24	Negative		negative	
2021-03-24	Negative		negative	
2021-03-24	Negative		negative	
2021-03-24	Negative		negative	
2021-03-24	Negative		negative	
2021-03-24	Negative		negative	
2021-03-24	Negative		negative	
2021-03-24	Negative		negative	
2021-03-24	Negative		negative	
2021-03-24	Negative		negative	
2021-03-24	Negative		negative	

	2021-03-24	Negative		negative	
	2021-03-24	Negative		negative	
	2021-03-24	Negative		negative	
	2021-03-24	Negative		negative	
	2021-03-24	Negative		negative	
	2021-03-24	Negative		negative	
	2021-03-24	Negative		negative	
	2021-03-24	Negative		negative	
	2021-03-24	Negative		negative	
	2021-03-24	Negative		negative	
	2021-03-24	Negative		negative	
	2021-03-24	Negative		negative	
	2021-03-24	Negative		negative	
	2021-03-24	Negative		negative	
	2021-03-24	Negative		negative	
	2021-03-24	Negative		negative	
	2021-03-24	Negative		negative	
	2021-03-24	Negative		negative	
	2021-03-24	Negative		negative	
	2021-03-24	Negative		negative	
	2021-03-24	Negative		negative	
	2021-03-24	Negative		negative	
	2021-03-24	Negative		negative	
	2021-03-24	Negative		negative	
	2021-03-24	Negative		negative	
	2021-03-24	Negative		negative	
	2021-03-24	Negative		negative	
	2021-03-24	Negative		negative	
	2021-03-24	Negative		negative	
	2021-03-24	Negative		negative	
	2021-03-24	Negative		negative	
	2021-03-24	Negative		negative	
	2021-03-24	Negative		negative	
	2021-03-24	Negative		negative	
	2021-03-24	Negative		negative	
	2021-03-24	Negative		negative	
	2021-03-24	Negative		negative	
	2021-03-24	Negative		negative	
	2021-03-24	Negative		negative	

	2021-03-24	Negative		negative	
	2021-03-24	Negative		negative	
	2021-03-24	Negative		negative	
	2021-03-24	Negative		negative	
	2021-03-24	Negative		negative	
	2021-03-24	Negative		negative	
	2021-03-24	Negative		negative	
	2021-03-24	Negative		negative	
	2021-03-24	Negative		negative	
	2021-03-24	Negative		negative	
	2021-03-24	Negative		negative	
	2021-03-24	Negative		negative	
	2021-03-24	Negative		negative	
	2021-03-24	Negative		negative	
	2021-03-24	Negative		negative	
	2021-03-24	Negative		negative	
	2021-03-24	Negative		negative	
	2021-03-24	Negative		negative	
	2021-03-24	Negative		negative	
	2021-03-24	Negative		negative	
	2021-03-24	Negative		negative	
	2021-03-24	Negative		negative	
	2021-03-24	Negative		negative	
	2021-03-24	Negative		negative	
	2021-03-24	Negative		negative	
	2021-03-24	Negative		negative	
	2021-03-24	Negative		negative	
	2021-03-24	Negative		negative	
	2021-03-24	Negative		negative	
	2021-03-24	Negative		negative	
	2021-03-24	Negative		negative	
	2021-03-24	Negative		negative	
	2021-03-24	Negative		negative	
	2021-03-24	Negative		negative	
	2021-03-24	Negative		negative	
	2021-03-24	Negative		negative	
	2021-03-24	Negative		negative	
	2021-03-24	Negative		negative	
	2021-03-24	Negative		negative	

		2021-03-24	Negative		negative	
		2021-03-24	Negative		negative	
		2021-03-24	Negative		negative	
		2021-03-24	Negative		negative	
		2021-03-24	Negative		negative	
		2021-03-24	Negative		negative	
		2021-03-24	Negative		negative	
		2021-03-24	Negative		negative	
		2021-03-24	Negative		negative	
		2021-03-24	Negative		negative	
		2021-03-24	Negative		negative	
		2021-03-24	Negative		negative	
		2021-03-24	Negative		negative	
		2021-03-24	Negative		negative	
		2021-03-24	Negative		negative	
		2021-03-24	Negative		negative	
		2021-03-24	Negative		negative	
		2021-03-24	Negative		negative	
		2021-03-24	Negative		negative	
		2021-03-24	Negative		negative	
		2021-04-06	Negative		negative	
		2021-04-06	Negative		negative	
		2021-04-06	Negative		negative	
		2021-04-06	Negative		negative	
		2021-04-06	Negative		negative	
		2021-04-06	Negative		negative	
		2021-04-06	Negative		negative	
		2021-04-06	Negative		negative	
		2021-04-06	Negative		negative	
		2021-04-06	Negative		negative	
		2021-04-06	Negative		negative	
		2021-04-06	Negative		negative	
		2021-04-06	Negative		negative	
		2021-04-06	Negative		negative	
		2021-04-06	Negative		negative	
		2021-04-06	Negative		negative	
		2021-04-06	Negative		negative	
		2021-04-06	Negative		negative	
		2021-04-06	Negative		negative	

	2021-04-06	Negative		negative	
	2021-04-06	Negative		negative	
	2021-04-06	Negative		negative	
	2021-04-06	Negative		negative	
	2021-04-06	Negative		negative	
	2021-04-06	Negative		negative	
	2021-04-06	Negative		negative	
	2021-04-06	Negative		negative	
	2021-04-06	Negative		negative	
	2021-04-06	Negative		negative	
	2021-04-06	Negative		negative	
	2021-04-06	Negative		negative	
	2021-04-06	Negative		negative	
	2021-04-06	Negative		negative	
	2021-04-06	Negative		negative	
	2021-04-06	Negative		negative	
	2021-04-06	Negative		negative	
	2021-04-06	Negative		negative	
	2021-04-06	Negative		negative	
	2021-04-06	Negative		negative	
	2021-04-06	Negative		negative	
	2021-04-06	Negative		negative	
	2021-04-06	Negative		negative	
	2021-04-06	Negative		negative	
	2021-04-06	Negative		negative	
	2021-04-06	Negative		negative	
	2021-04-06	Negative		negative	
	2021-04-06	Negative		negative	
	2021-04-06	Negative		negative	
	2021-04-06	Negative		negative	
	2021-04-06	Negative		negative	
	2021-04-06	Negative		negative	
	2021-04-06	Negative		negative	
	2021-04-06	Negative		negative	
	2021-04-06	Negative		negative	
	2021-04-06	Negative		negative	
	2021-04-06	Negative		negative	
	2021-04-06	Negative		negative	
	2021-04-06	Negative		negative	

	2021-04-06	Negative		negative	
	2021-04-06	Negative		negative	
	2021-04-06	Negative		negative	
	2021-04-06	Negative		negative	
	2021-04-06	Negative		negative	
	2021-04-06	Negative		negative	
	2021-04-06	Negative		negative	
	2021-04-06	Negative		negative	
	2021-04-06	Negative		negative	
	2021-04-06	Negative		negative	
	2021-04-06	Negative		negative	
	2021-04-06	Negative		negative	
	2021-04-06	Negative		negative	
	2021-04-06	Negative		negative	
	2021-04-06	Negative		negative	
	2021-04-06	Negative		negative	
	2021-04-06	Negative		negative	
	2021-04-06	Negative		negative	
	2021-04-06	Negative		negative	
	2021-04-06	Negative		negative	
	2021-04-06	Negative		negative	
	2021-04-06	Negative		negative	
	2021-04-06	Negative		negative	
	2021-04-06	Negative		negative	
	2021-04-06	Negative		negative	
	2021-04-06	Negative		negative	
	2021-04-06	Negative		negative	
	2021-04-06	Negative		negative	
	2021-04-06	Negative		negative	
	2021-04-06	Negative		negative	
	2021-04-06	Negative		negative	
	2021-04-06	Negative		negative	
	2021-04-06	Negative		negative	
	2021-04-06	Negative		negative	
	2021-04-06	Negative		negative	
	2021-04-06	Negative		negative	
	2021-04-06	Negative		negative	
	2021-04-06	Negative		negative	
	2021-04-06	Negative		negative	

	2021-04-06	Negative		negative	
	2021-04-06	Negative		negative	
	2021-04-06	Negative		negative	
	2021-04-06	Negative		negative	
	2021-04-06	Negative		negative	
	2021-04-06	Negative		negative	
	2021-04-06	Negative		negative	
	2021-04-08	Negative		negative	
	2021-04-08	Negative		negative	
	2021-04-08	Negative		negative	
	2021-04-08	Negative		negative	
	2021-04-08	Negative		negative	
	2021-04-08	Negative		negative	
	2021-04-08	Negative		negative	
	2021-04-08	Negative		negative	
	2021-04-08	Negative		negative	
	2021-04-08	Negative		negative	
	2021-04-08	Negative		negative	
	2021-04-08	Negative		negative	
	2021-04-08	Negative		negative	
	2021-04-08	Negative		negative	
	2021-04-08	Negative		negative	
	2021-04-08	Negative		negative	
	2021-04-08	Negative		negative	
	2021-04-08	Negative		negative	
	2021-04-08	Negative		negative	
	2021-04-08	Negative		negative	
	2021-04-08	Negative		negative	
	2021-04-08	Negative		negative	
	2021-04-08	Negative		negative	
	2021-04-08	Negative		negative	
	2021-04-08	Negative		negative	
	2021-04-08	Negative		negative	
	2021-04-08	Negative		negative	
	2021-04-08	Negative		negative	
	2021-04-08	Negative		negative	
	2021-04-08	Negative		negative	
	2021-04-08	Negative		negative	
	2021-04-08	Negative		negative	

		2021-04-08	Negative		negative	
		2021-04-08	Negative		negative	
		2021-04-08	Negative		negative	
		2021-04-08	Negative		negative	
		2021-04-08	Negative		negative	
		2021-04-08	Negative		negative	
		2021-04-08	Negative		negative	
		2021-04-08	Negative		negative	
		2021-04-08	Negative		negative	
		2021-04-08	Negative		negative	
		2021-04-08	Negative		negative	
		2021-04-08	Negative		negative	
		2021-04-08	Negative		negative	
		2021-04-08	Negative		negative	
		2021-04-08	Negative		negative	
		2021-04-08	Negative		negative	
		2021-04-08	Negative		negative	
		2021-04-08	Negative		negative	
		2021-04-08	Negative		negative	
		2021-04-08	Negative		negative	
		2021-04-08	Negative		negative	
		2021-04-08	Negative		negative	
		2021-04-08	Negative		negative	
		2021-04-08	Negative		negative	
		2021-04-08	Negative		negative	
		2021-04-08	Negative		negative	
		2021-04-08	Negative		negative	
		2021-04-08	Negative		negative	
		2021-04-08	Negative		negative	
		2021-04-08	Negative		negative	
		2021-04-08	Negative		negative	
		2021-04-08	Negative		negative	
		2021-04-08	Negative		negative	
		2021-04-08	Negative		negative	
		2021-04-08	Negative		negative	
		2021-04-08	Negative		negative	
		2021-04-08	Negative		negative	
		2021-04-08	Negative		negative	
		2021-04-08	Negative		negative	

	2021-04-08	Negative		negative	
	2021-04-08	Negative		negative	
	2021-04-08	Negative		negative	
	2021-04-08	Negative		negative	
	2021-04-08	Negative		negative	
	2021-04-08	Negative		negative	
	2021-04-08	Negative		negative	
	2021-04-08	Negative		negative	
	2021-04-08	Negative		negative	
	2021-04-08	Negative		negative	
	2021-04-08	Negative		negative	
	2021-04-08	Negative		negative	
	2021-04-08	Negative		negative	
	2021-04-08	Negative		negative	
	2021-04-08	Negative		negative	
	2021-04-08	Negative		negative	
	2021-04-08	Negative		negative	
	2021-04-08	Negative		negative	
	2021-04-08	Negative		negative	
	2021-04-08	Negative		negative	
	2021-04-08	Negative		negative	
	2021-04-08	Negative		negative	
	2021-04-08	Negative		negative	
	2021-04-08	Negative		negative	
	2021-04-08	Negative		negative	
	2021-04-08	Negative		negative	
	2021-04-08	Negative		negative	
	2021-04-08	Negative		negative	
	2021-04-08	Negative		negative	
	2021-04-08	Negative		negative	
	2021-04-08	Negative		negative	
	2021-04-08	Negative		negative	
	2021-04-08	Negative		negative	
	2021-04-08	Negative		negative	
	2021-04-08	Negative		negative	
	2021-04-08	Negative		negative	
	2021-04-08	Negative		negative	
	2021-04-08	Negative		negative	
	2021-04-08	Negative		negative	

		2021-04-08	Negative		negative	
		2021-04-08	Negative		negative	
		2021-04-08	Negative		negative	
		2021-04-08	Negative		negative	
		2021-04-08	Negative		negative	
		2021-04-08	Negative		negative	
		2021-04-08	Negative		negative	
		2021-04-08	Negative		negative	
		2021-04-08	Negative		negative	
		2021-04-08	Negative		negative	
		2021-04-08	Negative		negative	
		2021-04-08	Negative		negative	
		2021-04-08	Negative		negative	
		2021-04-08	Negative		negative	
		2021-04-08	Negative		negative	
		2021-04-08	Negative		negative	
		2021-04-08	Negative		negative	
		2021-04-08	Negative		negative	
		2021-04-08	Negative		negative	
		2021-04-08	Negative		negative	
		2021-04-08	Negative		negative	
		2021-04-08	Negative		negative	
		2021-04-08	Negative		negative	
		2021-04-08	Negative		negative	
		2021-04-08	Negative		negative	
		2021-04-08	Negative		negative	
		2021-04-08	Negative		negative	
		2021-04-08	Negative		negative	
