# Supplementary material for: Comprehensive analytical and clinical evaluation of a RNA extraction-free saliva-based molecular assay for SARS-CoV-2
Source: PLoS One. 2022 May 5;17(5):e0268082. doi: 10.1371/journal.pone.0268082 (PMC9070935; doi:10.1371/journal.pone.0268082)
Supplement: S3 Table — (PDF) [file pone.0268082.s003.pdf]

quadruplex qPCR Clinical Validation

#	saliva code	quadruplex qPCR	matched MHS sample
1	300551	**15,9**	19,77
2	300621	**17,9**	20,74
3	301002	**19**	18,13
4	300535	**19,1**	19,52
5	300600	**20,3**	24,62
6	300564	**20,7**	18,88
7	300984	**21,8**	22,38
8	300884	**22,2**	19,27
9	300517	**22,5**	16,83
10	300969	**23,5**	17,5
11	300913	**23,9**	15,69
12	300947	**24**	15,43
13	300574	**24,2**	18,52
14	300536	**24,4**	19,69
15	300593	**24,5**	17,54
16	300971	**24,7**	17,71
17	300583	**24,8**	19,38
18	300889	**25,1**	19,95
19	300897	**25,7**	15,03
20	300575	**25,9**	14,64
21	300992	**26**	28,12
22	300608	**26,2**	16,01
23	300622	**26,7**	19,92
24	300939	**27**	24,93
25	300531	**27,2**	20,43
26	300617	**27,2**	20,94
27	300945	**27,6**	19,19
28	301000	**27,9**	20,54
29	300905	**28**	17,43
30	300582	**28,4**	28,11
31	300933	**28,9**	18,86
32	300604	**29,4**	18,1
33	300592	**29,6**	21,9
34	300899	**29,7**	17,57
35	300514	**29,7**	29,07
36	300904	**30,1**	24,9
37	300540	**31,1**	18,91
38	301001	**31,2**	28,29
39	300534	**31,3**	17,46
40	300926	**32,1**	20,59
41	300977	**32,6**	16,89
42	300934	**33,5**	36,61
43	300924	**34,1**	29,32
44	300929	**neg**	22,98
45	300927	**neg**	26,24
46	300507	**neg**	neg
47	300509	**neg**	neg
48	300510	**neg**	neg
49	300513	**neg**	neg
50	300516	**neg**	neg
51	300519	**neg**	neg
52	300520	**neg**	neg
53	300521	**neg**	neg
54	300525	**neg**	neg
55	300526	**neg**	neg

56	300528	**neg**	neg
57	300529	**neg**	neg
58	300532	**neg**	neg
59	300538	**neg**	neg
60	300539	**neg**	neg
61	300541	**neg**	neg
62	300542	**neg**	neg
63	300543	**neg**	neg
64	300544	**neg**	neg
65	300545	**neg**	neg
66	300548	**neg**	neg
67	300550	**neg**	neg
68	300554	**neg**	neg
69	300555	**neg**	neg
70	300556	**neg**	neg
71	300557	**neg**	neg
72	300559	**neg**	neg
73	300560	**neg**	neg
74	300561	**neg**	neg
75	300563	**neg**	neg
76	300565	**neg**	neg
77	300566	**neg**	neg
78	300567	**neg**	neg
79	300569	**neg**	neg
80	300570	**neg**	neg
81	300571	**neg**	neg
82	300572	**neg**	neg
83	300573	**neg**	neg
84	300576	**neg**	neg
85	300577	**neg**	neg
86	300579	**neg**	neg
87	300580	**neg**	neg
88	300581	**neg**	neg
89	300584	**neg**	neg
90	300585	**neg**	neg
91	300587	**neg**	neg
92	300588	**neg**	neg
93	300589	**neg**	neg
94	300590	**neg**	neg
95	300591	**neg**	neg
96	300595	**neg**	neg
97	300596	**neg**	neg
98	300598	**neg**	neg
99	300599	**neg**	neg
100	300601	**neg**	neg
101	300603	**neg**	neg
102	300605	**neg**	neg
103	300606	**neg**	neg
104	300609	**neg**	neg
105	300610	**neg**	neg
106	300611	**neg**	neg
107	300612	**neg**	neg
108	300613	**neg**	neg
109	300614	**neg**	neg
110	300615	**neg**	neg
111	300616	**neg**	neg
112	300618	**neg**	neg
113	300620	**neg**	neg
114	300623	**neg**	neg

115	300625	**neg**	neg
116	300626	**neg**	neg
117	300627	**neg**	neg
118	300878	**neg**	neg
119	300880	**neg**	neg
120	300882	**neg**	neg
121	300883	**neg**	neg
122	300886	**neg**	neg
123	300887	**neg**	neg
124	300888	**neg**	neg
125	300890	**neg**	neg
126	300892	**neg**	neg
127	300893	**neg**	neg
128	300894	**neg**	neg
129	300895	**neg**	neg
130	300896	**neg**	neg
131	300901	**neg**	neg
132	300902	**neg**	neg
133	300903	**neg**	neg
134	300906	**neg**	neg
135	300907	**neg**	neg
136	300909	**neg**	neg
137	300910	**neg**	neg
138	300911	**neg**	neg
139	300912	**neg**	neg
140	300914	**neg**	neg
141	300916	**neg**	neg
142	300918	**neg**	neg
143	300920	**neg**	neg
144	300921	**neg**	neg
145	300922	**neg**	neg
146	300923	**neg**	neg
147	300925	**neg**	neg
148	300928	**neg**	neg
149	300930	**neg**	neg
150	300932	**neg**	neg
151	300935	**neg**	neg
152	300936	**neg**	neg
153	300937	**neg**	neg
154	300938	**neg**	neg
155	300940	**neg**	neg
156	300941	**neg**	neg
157	300942	**neg**	neg
158	300943	**neg**	neg
159	300944	**neg**	neg
160	300946	**neg**	neg
161	300948	**neg**	neg
162	300949	**neg**	neg
163	300950	**neg**	neg
164	300951	**neg**	neg
165	300952	**neg**	neg
166	300954	**neg**	neg
167	300955	**neg**	neg
168	300956	**neg**	neg
169	300957	**neg**	neg
170	300958	**neg**	neg
171	300961	**neg**	neg
172	300962	**neg**	neg
173	300964	**neg**	neg

174	300965	**neg**	neg
175	300966	**neg**	neg
176	300967	**neg**	neg
177	300970	**neg**	neg
178	300972	**neg**	neg
179	300973	**neg**	neg
180	300974	**neg**	neg
181	300975	**neg**	neg
182	300976	**neg**	neg
183	300978	**neg**	neg
184	300979	**neg**	neg
185	300980	**neg**	neg
186	300981	**neg**	neg
187	300982	**neg**	neg
188	300983	**neg**	neg
189	300985	**neg**	neg
190	300986	**neg**	neg
191	300987	**neg**	neg
192	300988	**neg**	neg
193	300989	**neg**	neg
194	300990	**neg**	neg
195	300991	**neg**	neg
196	300994	**neg**	neg
197	300996	**neg**	neg
198	300998	**neg**	neg
